# Malnutrition prevalence and associated biochemical factors among drug-resistance tuberculosis (DR-TB) patients at key treatment sites in Conakry City, Republic of Guinea

**DOI:** 10.11604/pamj.2021.38.279.27270

**Published:** 2021-03-17

**Authors:** Aboubacar Sidiki Magassouba, Almamy Amara Touré, Boubacar Djelo Diallo, Lansana Mady Camara, Demba Touré, Nfanly Conté, Macka Diaby, Soriba Naby Camara, Gnoume Camara, Adama Marie Bangoura, Tamba Alima Kamano, Adrien Fapeingou Tounkara

**Affiliations:** 1 National Tuberculosis Control Program, International Union Against Tuberculosis and Lung Disease, Guinea,; 2 Centre National de Formation et de Recherche en Santé Rurale de Maferinyah, Forecariah, Guinea,; 3 Faculty of Health Science and Technology, Gamal Abdel Nasser University of Conakry, Conakry, Guinea,; 4 Faculty of Health Science and Technology, Gamal Abdel Nasser University of Conakry, Conakry, Guinea,; 5 Pneumo Physiology Department, Conakry University Hospital Ignace Deen, Conakry, Guinea,; 6 National Tuberculosis Control Program, Guinea,; 7 Department of Sociology of the University of Sonfonia, Conakry, Guinea,; 8 National AIDS and Hepatitis Control Program, Guinea

**Keywords:** Drug-resistant tuberculosis, malnutrition, associated factors, Guinea

## Abstract

**Introduction:**

drug-resistant tuberculosis is a major global health problem and a threat to health security given the increase in the number of cases and the challenges associated with care. Besides, the relationship between poor nutritional status and tuberculosis is clearly established. For relevant and evidence-based public health decision-making regarding the management of malnutrition in patients with drug-resistant tuberculosis in the initial phase, it is essential to estimate the prevalence of malnutrition and understand the risk factors associated with it.

**Methods:**

we performed a retrospective cohort study in drug-resistant tuberculosis patients aged 18 years and older, among which the nutritional status was assessed through BMI. All predictors were included in a prediction model using the multivariate logistic model according to the lowest Akaike criterion. Discrimination and model calibration was evaluated using receiver performance analysis, and the Hosmer and Lemeshow test.

**Results:**

this study revealed a prevalence of malnutrition of 64.7% in drug-resistant tuberculosis patients in our 218-patient series. The factors associated with malnutrition were: unsuccessful treatment, the active presence of mycobacterium tuberculosis, increased bacteriological conversion time, increased serum creatinine, increased transaminase SGPT of the liver, and anaemia. Some of the factors not associated with malnutrition included the history of anti-tuberculosis treatment, vomiting, hepatic SGPT, initial AFB count, smear and culture conversion time, depression, and chest X-ray.

**Conclusion:**

malnutrition remains a concern among drug-resistant tuberculosis patients in Guinea as it affects more than half of them with a negative impact on the outcome of treatment. Implementing specific interventions for these high-risk patients, including nutritional supplementation, psychosocial support, and treatment for tuberculosis, can improve management for better treatment outcomes.

## Introduction

Guinea is ranked among the countries with a high incidence of tuberculosis and TB/HIV coinfection, both of which are major causes of morbidity and mortality [1]. Even though incidence and mortality are steadily declining in Guinea, the tuberculosis epidemic remains a concern because of the increasing occurrence of drug-resistant forms and the poor control of comorbidities. The country is now experiencing the emergence of forms of resistance to major molecules for first-line treatment such as rifampicin and isoniazid. This is reflected in the increase in the number of cases of DR-TB detected, from 178 cases in 2016 to 265 cases in 2018 [2]. Among people infected with TB, 10-15% would develop the disease if they do not receive adequate antituberculosis treatment [3]. This risk is much higher for those with a compromised immune system, such as people living with HIV, who are malnourished or have diabetes [4]. Most DR-TB patients screened in Guinea have a longer therapeutic route (patients already treated for susceptible tuberculosis), as the Xpert® MTB/RIF test is not first-line for all patients.

Tuberculosis and malnutrition are linked in a complicated relationship that has been known for a long time. Infection can lead to undernutrition associated with increased metabolic demand and decreased intake, and nutritional deficiencies can aggravate the disease or delay healing by reducing essential immune functions [5]. Malnutrition is defined as the deficiency, excess, or imbalance in the energy and/or nutritional intake of a person [6]. This medical condition may support the disease in the direction of aggravation and lack of satisfactory therapeutic results, which is why it is essential to identify these comorbidities in people with tuberculosis to ensure early diagnosis and to improve management [7]. Most people with active TB experience weight loss that can be caused by several factors, including decreased dietary intake due to loss of appetite, nausea, and abdominal pain [8].

In 2015, according to the European Society of Clinical Nutrition, the diagnosis of malnutrition was based on a low body mass index (BMI), defined as less than <18.5 kg/m^2^, or a combination of weight loss and low mass free fat or low BMI (<20 kg/m^2^ or <22 kg/m^2^, depending on age). The same guideline is used by the WHO to identify malnutrition [9], although malnutrition has been documented in patients with high BMI [10, 11]. The body mass index at the beginning of tuberculosis treatment is a statistical nutritional risk factor frequently used in tuberculosis research [7]. However, a change in body weight after the start of TB treatment may be indicative of improvement or worsening of nutritional status during TB treatment [8].

The socioeconomic status of the patient is an element to be considered during the initiation of antituberculosis treatment. According to the 2012 DHS [12], concerning malnutrition in the population, significant disparities exist depending on the living environment and economic status. The analysis of the association between malnutrition and DR-TB could provide useful information for decision-making in the management and prevention of nutrition in patients. Our study aimed to determine the prevalence of malnutrition and to identify the associated factors in DR-TB patients during the initial phase of treatment. To our knowledge, no study has been conducted on this subject in Guinea.

## Methods

**Diagnosis of DR-TB, tuberculosis, and malnutrition:** according to the technical guide of the tuberculosis control program, diagnosis of DR-TB cases is based on careful clinical examination, bacteriological examination (direct examination of sputum for AFB research), XpertÂ® MTB/RIF, culture and susceptibility testing of the bacillary strain against antituberculosis), these examinations are supplemented by radiography for the detection of lesions that could be associated and a pre-therapeutic assessment to examine the patient's ability to receive second-line antituberculosis treatment. The body mass index (BMI) was used to assess the nutritional status of patients. With the metric system, the formula for BMI is weight in kilograms divided by height in square meters. For adults, the BMI is interpreted using the standard weight status categories. These categories are the same for people of all types and ages [13]. Measurements of weight (in kilograms) and height (in centimeters) were made using a medical scale and an appropriate adult measuring rod, respectively. These devices are available at DR-TB treatment sites.

**Study setting and population:** we analyzed surveillance data from 218 patients with DR-TB enrolled between June 7, 2016, and June 22, 2018, in a multicenter longitudinal cohort study conducted in three large centres for DR-TB in Conakry (Ignace-Deen, Carrière, and Tombolia). All patients were initially seen and followed monthly for a period of 9 months, according to the WHO standardized treatment regimen guidelines of 9 to 12 months [14]. Patients younger than 18 years old were excluded from the analysis.

**Data collection:** the data were collected from March 2^nd^ to July 12^th^, 2020, using a case report form (CRF) from the DR-TB register containing information on sociodemographic and clinical characteristics, laboratory test results (sputum smear or culture) and radiography for all registered patients in treatment centres. Additional information was collected through patient medical records and results reports. The following clinical, paraclinical, and demographic data were extracted: age, sex, residence, comorbidity, HIV status, history of previously treated tuberculosis, presence of caves on the chest X-ray as determined by the lead radiologist, baseline data on weight, height for calculation of BMI, sputum smear and culture, clinical symptoms (chest pain, cough, night sweats, nausea, vomiting) and biological data (haemoglobin, SGPT, SGOT, creatinine level, number of leukocytes, number of platelets, number of lymphocytes). Moreover, we extracted the depression status when the patient had to indicate whether he was depressed or anxious.

**Outcome measures and selection of predictors:** for the calculation of prevalence, a distinction was made between underweight (<18.5kg/m^2^), ideal weight (18.5-25kg/m^2^), excess weight (26-30kg/m^2^) and obesity (>30kg/m^2^). However, for reasons of focused analysis on malnutrition, we categorized the variable into two groups by grouping the ideal weight and obesity as a reference category, in addition to the underweight category. Potential predictors are contributed by sociodemographic predictors (gender, age), clinical (cough, dyspnea, chest pain, night sweats, nausea, vomiting, HIV status, history of TB treatment, depression, adherence, treatment outcome) and paraclinical (number of colonies by initial smear, initial number of colonies, initial culture, X-ray lung cavities, number of leukocytes, number of haemoglobin, number of platelets, number of lymphocytes, number of creatinine, liver SGOT, SGPT liver, smear conversion delay, culture conversion delay).

**Statistical analysis:** frequencies (per cent) or means (standard deviation; SD) were used to describe categorical and continuous variables. Univariate logistic regression was used to identify prognostic factors related to treatment with malnutrition, and then candidates with a p-value less than 0.10 were entered into the multivariate logistic regression. The independent predictors of malnutrition identified were selected using a backward procedure based on the lowest Akaike information criterion. Odds ratio (OR) with 95% confidence intervals (95% CI) were used as association parameters and the significance level was p less than 5%. The assumption of risk proportionality and log-linearity has been verified.

The Hosmer and Lemeshow fit validity test was used to measure the extent to which the probabilities predicted by the model correspond to the probabilities observed. Moreover, a calibration plot of observed and predicted probabilities was performed. An analysis of the operating characteristics of the receiver (ROC) by applying the Youden index method [15], to obtain the optimal score of the cutoff point was used to test the discriminating performances of our model. Then, at this optimal threshold, performance measures, including sensitivity, specificity, and positive and negative (PV) predictive values were estimated. The final model was validated internally using the 1000 bootstrap sample procedure. All analyses described above were performed with R version 3.5.1 software.

**Ethics approval and consents to participate:** the Cardio-pneumology Chair, attached to the Faculty of Medicine of the Gamal Abdel Nasser University in Conakry, approved the protocol for this study (023/CCP/18) which had also received administrative authorization from the Ministry of Health of Guinea via the National Tuberculosis Control Program (PNLAT). Patient data has been anonymized and confidentiality is guaranteed in accordance with the Declaration of Helsinki. The ethics approval process conforms to national regulations for such research.

## Results

**Description of demographic and clinical characteristics:** our patients were predominantly male (68.3%), of whom 61% were malnourished, while women (31.6%) were women with a much higher proportion of malnourished women (72.5%). The average age of patients was 33.7, with 95% CI [32.2-35.2], most live in an urban setting (77.5%). HIV was positive in 50 patients, 36 (72%) of whom had a BMI <18.5. According to the category of patients, 38 patients (17.4%) were new DR-TB cases, and 180 patients (82.6%) had already received first-line anti-TB treatment. The most common clinical signs were cough (94%), night sweats (55%), chest pain (60.5%), and dyspnea (31.2%), and depression was seen in only (4.6%) patients. The cavity on chest radiograph was present in (16.9%) patients, and 72.9% of our patients had led their successful treatment at the end of follow-up (Table 1).

**Table 1:** basic socio-demographic and clinical characteristics

Variables		Gender		Total (%)
		Female	Male	
Age	Mean (SD)	33.1 (10.3)	34.0 (11.8)	33.7 (11.3)
BMI	<=18.5	50 (72.5)	91 (61.1)	141 (64.7)
	>18.5	19 (27.5)	58 (38.9)	77 (35.3)
Residence	Urban	53 (76.8)	116 (77.9)	169 (77.5)
	Rural	16 (23.2)	33 (22.1)	49 (22.5)
Years of inclusion	2016	5 (7.2)	13 (8.7)	18 (8.3)
	2017	40 (58.0)	96 (64.4)	136 (62.4)
	2018	24 (34.8)	40 (26.8)	64 (29.4)
HIV status	Negative	41 (59.4)	127 (85.2)	168 (77.1)
	Positive	28 (40.6)	22 (14.8)	50 (22.9)
History TB treatment	New DR-TB case	22 (31.9)	16 (10.7)	38 (17.4)
	Previous case treated	47 (68.1)	133 (89.3)	180 (82.6)
AFB count initial smear	<=3	43 (62.3)	72 (48.3)	115 (52.8)
	>=3	26 (37.7)	77 (51.7)	103 (47.2)
AFB count initial culture	<=3	30 (43.5)	62 (41.6)	92 (42.2)
	>=3	39 (56.5)	87 (58.4)	126 (57.8)
Lung Cavities X-Ray	No	62 (89.9)	129 (86.6)	191 (87.6)
	yes	7 (10.1)	20 (13.4)	27 (12.4)
Cough	No	4 (5.8)	9 (6.0)	13 (6.0)
	Yes	65 (94.2)	140 (94.0)	205 (94.0)
Dyspnea	No	47 (68.1)	103 (69.1)	150 (68.8)
	Yes	22 (31.9)	46 (30.9)	68 (31.2)
Chest Pain	No	40 (58.0)	92 (61.7)	132 (60.6)
	Yes	29 (42.0)	57 (38.3)	86 (39.4)
Night Sweat	No	26 (37.7)	72 (48.3)	98 (45.0)
	Yes	43 (62.3)	77 (51.7)	120 (55.0)
Nausea	No	66 (95.7)	137 (91.9)	203 (93.1)
	Yes	3 (4.3)	12 (8.1)	15 (6.9)
Vomiting	No	65 (94.2)	131 (87.9)	196 (89.9)
	Yes	4 (5.8)	18 (12.1)	22 (10.1)
Leukocytes count	Mean (SD)	8.2 (5.5)	7.9 (2.9)	8.0 (3.9)
Haemoglobin count	Mean (SD)	10.0 (2.1)	10.9 (2.2)	10.7 (2.2)
Platelets count	Mean (SD)	71.9 (153.9) 3	92.5 (163.7) 3	86.0 (160.6)
Lymphocytes count	Mean (SD)	1.8 (1.0)	1.8 (1.4)	1.8 (1.3)
Creatinine count	Mean (SD)	75.0 (18.8)	80.7 (19.3)	78.9 (19.3)
Liver SGOT	Mean (SD)	29.8 (5.7)	29.1 (4.9)	29.3 (5.2)
Liver SGPT	Mean (SD)	30.9 (5.0)	32.3 (5.1)	31.9 (5.1)
Time to smear convert	Mean (SD)	50.0 (26.8)	58.7 (33.1)	56.0 (31.5)
Time to culture convert	Mean (SD)	46.6 (26.5)	48.5 (22.6)	47.9 (23.9)
Treatment outcome	Unsuccessful	24 (34.8)	35 (23.5)	59 (27.1)
	Successful	45 (65.2)	114 (76.5)	159 (72.9)
Depression	No	63 (91.3)	145 (97.3)	208 (95.4)
	Yes	6 (8.7)	4 (2.7)	10 (4.6)
SD = standard deviation; n = number; % = percentage; BMI = body mass index				

**Figure 1:**
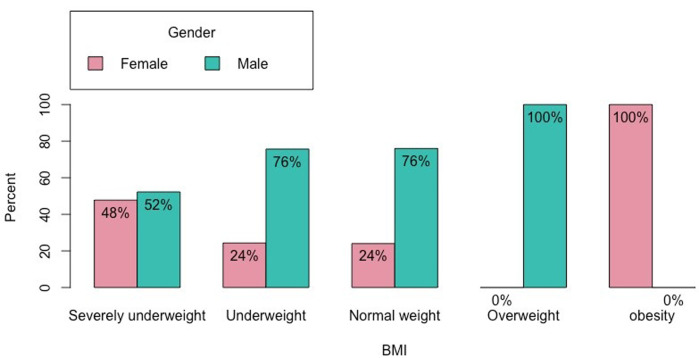
distribution of patients by weight status at initiation of second-line anti-TB treatment

**Prevalence of malnutrition:** of the 218 participants in the study, 141 (64.7%) had a BMI of less than 18.5 and 77 (35.3%) had a BMI greater than 18.5. In classifying malnutrition according to the European Society of Clinical Nutrition and Metabolism, we note that 75 (34.4%) were of average weight, 74 (33.9%) were very underweight, 67 (30.7) had an underweight and one overweight patient (0.5%). Men were the most affected by severe underweight, with a proportion of 16% of the total against 14.7% for women and 25.7% against 8.3%, respectively, for the moderate weight (Figure 1).

**Predictors of malnutrition:** a univariate logistic regression (Table 2) found the factors potentially associated with malnutrition. After inclusion of all candidate variables in the univariate regression model, the decrease in hemoglobin (OR = 1.2[1.04-1.3, p = 0.01]), the presence of night sweats (OR = 0.5[0.3-0.9, p = 0.02]), the presence of cough (OR = 7.1[1.3-130.1, p = 0.06]) were the factors statistically associated with malnutrition. However, several other variables including sex, nausea, vomiting, leukocyte count, creatinine, and SGPT had p values <= 0.1. These candidate variables were included in the multivariate regression model (Table 2) and the predictors were selected through the backward procedure based on the lowest Akaike information criterion (AIC = 258.2). Thus, the final model (Table 3) identified as predictors of malnutrition: the unsuccessful treatment (OR = 3.8[1.7-8.9, p = 0.001]), the active presence of the mycobacterium tuberculosis characterized by the presence of cough (OR =32.8 [4.61-706.4, p = 0.003]), night sweats (OR =0.3[0.1-0.5, p <0.001]), the presence of cavity on chest X-ray (OR =3.7[1.4-10.6, p = 0.012]), and the increase in bacteriological conversion time (OR =0.99 [0.97-1.00, p = 0.014]). And, the increase in serum creatinine (OR = 1.02 [1.00-1.04, p = 0.036]), increase in liver SGPT transaminase (OR = 0.9[0.8-0.9, p = 0.02]) and anemia (OR = 1.1[0.9-1.3, p = 0.083]).

**Table 2:** predictors of malnutrition among DR-TB patients in Guinea, uni, and multivariate logistic regression analysis

Predictors	OR univariable (95% IC)	P-value	OR multivariable (95% IC)	P-value
Gender (Male vs Female)	1.68 (0.91-3.18)	0.103	1.76 (0.80-3.98)	0.166
Age	0.99 (0.96-1.01)	0.329	0.98 (0.95-1.01)	0.228
Residence (Urban vs rural)	0.86 (0.43-1.67)	0.657	0.62 (0.25-1.46)	0.279
Years of inclusion (2017 vs 2016)	2.10 (0.71-7.72)	0.212	1.79 (0.52-7.42)	0.382
Years of inclusion (2018 vs 2016)	1.83 (0.58-7.07)	0.332	2.08 (0.50-10.01)	0.331
HIV (Yes vs No)	0.65 (0.32-1.27)	0.219	0.74 (0.27-1.99)	0.557
History of TB treatment (Previously vs Newly)	1.22 (0.59-2.67)	0.596	0.70 (0.23-2.04)	0.512
AFB count initial smear (≥3+ vs <3 +)	1.05 (0.60-1.83)	0.860	1.10 (0.53-2.29)	0.803
AFB count initial culture (≥3+ vs <3 +)	1.50 (0.85-2.67)	0.166	1.63 (0.78-3.48)	0.199
Lung Cavities X Ray (Yes vs No)	1.19 (0.53-2.57)	0.670	3.06 (0.97-10.04)	0.058
Cough (Yes vs No)	7.07 (1.35-130.06)	0.063	35.70 (4.26-826.25)	0.004
Dyspnea (Yes vs No)	0.56 (0.29-1.03)	0.067	0.62 (0.27-1.41)	0.262
Chest Pain (Yes vs No)	0.9 7 (0.55-1.71)	0.913	1.30 (0.62-2.77)	0.491
Night sweat (Yes vs No)	0.51 (0.29-0.89)	0.018	0.27 (0.13-0.54)	P<0.001
Nausea (Yes vs No)	0.91 (0.27-2.66)	0.867	1.90 (0.34-11.66)	0.466
Vomiting (Yes vs No)	0.37 (0.11-1.05)	0.086	0.27 (0.05-1.15)	0.095
Leukocytes count (m ± SD)	0.91 (0.82-1.00)	0.065	0.89 (0.77-1.00)	0.080
Haemoglobin count (m ± SD)	1.21 (1.06-1.39)	0.007	1.06 (0.89-1.28)	0.513
Platelets count (m ± SD)	1.00 (1.00-1.00)	0.970	1.00 (1.00-1.00)	0.705
Lymphocytes count (m ± SD)	1.20 (0.99-1.53)	0.092	1.21 (0.93-1.70)	0.211
Creatinine count (m ± SD)	1.01 (1.00-1.03)	0.112	1.02 (1.00-1.04)	0.122
Liver SGOT (m ± SD)	0.99 (0.94-1.05)	0.806	1.03 (0.95-1.12)	0.426
Liver SGPT (m ± SD)	0.96 (0.91-1.02)	0.186	0.92 (0.85-0.99)	0.035
Time to smear convert (m ± SD)	0.99 (0.98-1.00)	0.125	0.99 (0.97-1.00)	0.049
Time to culture convert (m ± SD)	1.00 (0.99-1.01)	0.952	1.00 (0.99-1.02)	0.778
Treatment outcome (Unsuccessful vs Successful)	2.38 (1.22-4.92)	0.014	3.20 (1.32-8.33)	0.013
Depression (Yes vs No)	0.78 (0.16-2.88)	0.719	1.73 (0.29-8.81)	0.520

OR = odds ratio, CI = confidence interval, HIV = human immunodeficiency virus

**Table 3:** predictors of malnutrition among DR-TB patients selected in the final model of the multivariate regression

Predictors	Estimate	Std. Error	z value	Pr (>|z|)
(Intercept)	-4.311436	1.807937	-2.385	0.017092 *
Treatmentoutcome (Unsuccessful vs. successful)	1.234764	0.400429	3.084	0.002045 **
Cough (Yes vs. No)	3.560339	1.191524	2.988	0.002808 **
Creatinine count	0.019001	0.008806	2.158	0.030949 *
Liver SGPT	-0.075062	0.032983	-2.276	0.022858 *
Lung cavities X-Ray (Yes vs. No)	1.011918	0.523634	1.932	0.053299.
Night sweat (Yes vs No)	-1.258227	0.336029	-3.744	0.000181 ***
Time to smear convert	-0.013739	0.005678	-2.420	0.015527 *
Haemoglobin count	0.140811	0.076279	1.846	0.064892

Log Likelihood= -120.102, Akaike Inf. Crit.=258.205

**Figure 2:**
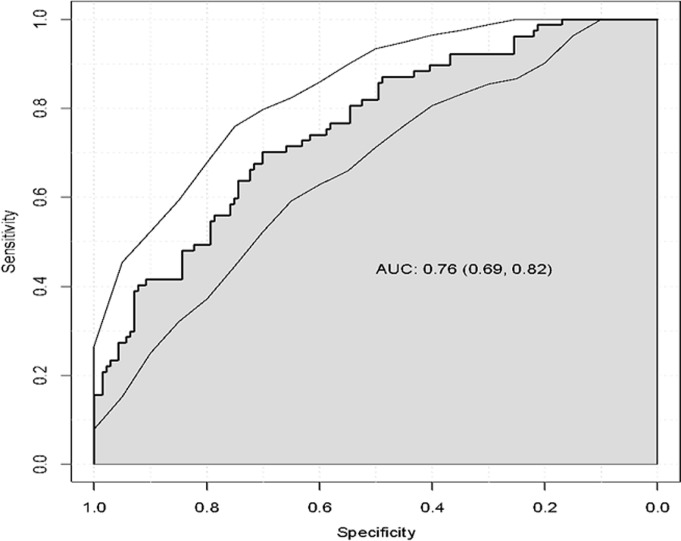
ROC-curve of the multivariate analyses; ROC-curve =receiver-operating characteristic; AUC = area under the curve; 95% CI = 95% confidence interval

**Figure 3:**
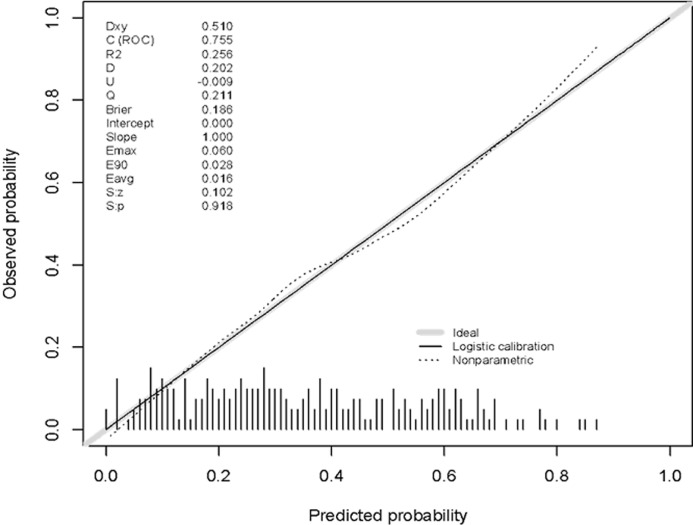
calibration plot of the predicted probability versus the observed probability

A characteristic operating curve of the receiver illustrates a good discriminatory capacity of our model (Figure 2), the index c adjusted to predict malnutrition was 0.76[95% CI, 0.69 - 0.82]. The optimism of 1000 samples was 0.018, which corresponds to a corrected value of the c-statistic with the optimism of 0.74. The p-value of 0.4 produced by the Hosmer and Lemeshow fit quality test confirms that the model is statistically adjusted with adequate discriminatory capacity (P> 0.05). Finally, the calibration curve (Figure 3) shows a good agreement between the risk of malnutrition estimated by the final model and the observed cases of malnutrition, across the predicted probability continuum.

## Discussion

This retrospective cohort study revealed a prevalence of malnutrition of 64.7% in tuberculosis patients on second-line treatment. We also note that 16% of men were severely malnourished, and 25.7% were underweight. A study in nine countries in West and Central Africa on the evaluation of the treatment results of patients on antituberculosis treatment regimens revealed a prevalence of 56 [16]. A similar prevalence (56%) has been reported by Elliot *et al*. in South Africa [17]. Another study by Nyaki FS *et al*. in Tanzania found a prevalence of 53% in Tanzania [18], unlike the research in Ethiopia, which reported a prevalence of only 10.2% [19]. At the same time, in East Asia, the Ho Park study in South Korea revealed 24.3% of malnutrition (BMI <18.5) [20] and Bei C 59.9% in China [21]. Our study population was relatively young and male 68.35%), with a prevalence of 64.7%, average age of patients 33.7, 95% CI [32.2, 35.2], resident most in urban areas. In Guinea, the same male predominance is also sawed in patients receiving first-line treatment.

The malnourished patients found in our study had active tuberculosis, which explained the persistence of night sweats from coughing and the presence of radiological lesions and was, for the most part, patients in retreatment. Our patients have the same profile as those described by other authors in Malawi [22], Tanzania [18], and South Africa [23]. Although several studies have shown that a low BMI is a risk factor for mortality in tuberculosis patients co-infected with HIV [23-25], this factor was not one of the identified predictors of our study. HIV was positive in 50 patients, 36 (72%) of whom had a BMI <18.5 but were not associated with malnutrition (P value> 0.05). The role of malnutrition, although recognized among the factors of non-therapeutic success, by several studies [17, 18, 26, 27], its management is still insufficient in several tuberculosis control programs due to lack of financial means. Our results are consistent with those of the literature according to which patients suffering from malnutrition were highly likely not to succeed in their antituberculosis treatment.

We found a hepatic transaminase (SGPT) disturbance during malnutrition (p = 0.02). Medicines used to treat tuberculosis can lead to high liver transaminases, which is a typical situation. The increases may be small, but hepatotoxicity can have serious consequences if left unattended [28-30]. Active bacillary tuberculosis is often associated with the most severe symptoms (fever, night sweats, cough, and chest pain); we have shown a link with malnutrition (p> 0.05). Infection can lead to undernutrition associated with increased metabolic demand and decreased intake, and nutritional deficiencies can worsen the disease or delay healing by reducing essential immune functions [5]. In a patient treated for active tuberculosis by second-line treatment, the occurrence of renal symptoms is common. Our study showed an increase in serum creatinine during malnutrition; this can be explained using second-line injectable antituberculosis drugs (aminoglycosides and capreomycin), known for their nephrotoxic and ototoxic effects [8, 31].

## Conclusion

Our study highlighted the factors associated with malnutrition during the first phase of treatment in the context of cause and effect. The relevant factors found are the presence of active Tb, increased hepatic transaminases, increased bacteriological conversion time, and decreased serum creatinine. The study also proved the negative effect of malnutrition on the success of TB treatment. Taking these factors into account in the clinical practice of TB patients by taking steps to improve nutritional intake in patients would improve their management.

### What is known about this topic

- Tuberculosis can lead to malnutrition, and malnutrition can still make tuberculosis worse if left untreated. Nutritional support for tuberculosis patients is increasingly becoming a common practice;
- Most people with active TB lose weight; weight gain during treatment is a sign of improvement in their condition;
- Systematic screening for tuberculosis in malnourished patients is an initiative that has been tested in several countries and recognized as good practice by WHO.

### What this study adds

- We retain from this study that malnutrition is widespread among multidrug-resistant tuberculosis patients in Guinea. These patients generally have a long course because they benefit from a late diagnosis in most cases in our context, where the Xpert MTB/RIF test and other tests offered by the WHO is only reserved for a small group of targets (cases of retreatement, HIV +, children, and prisoners);
- This study demonstrated a strong relationship between the deterioration of the nutritional status of multidrug-resistant tuberculosis patients and the deterioration of clinical characteristics (hemoglobin, active symptoms of tuberculosis, disturbance of renal workup and prolongation of the bacteriological conversion time). This justifies the legitimacy of the nutritional support that must be systematically provided to these patients to hope for compliance with treatment;
- This study also shows the need to immediately apply the WHO recommendations concerning the systematization of the Xpert MTB/RIF test as a first-line diagnosis and to put in place a bold policy of nutritional support for patients to improve on the one hand the condition of the patient clinic and the outcome of the treatment at term.
